# Assessing Chemical Constituents of *Mimosa caesalpiniifolia* Stem Bark: Possible Bioactive Components Accountable for the Cytotoxic Effect of *M. caesalpiniifolia* on Human Tumour Cell Lines 

**DOI:** 10.3390/molecules20034204

**Published:** 2015-03-05

**Authors:** Nayana Bruna Nery Monção, Bruno Quirino Araújo, Jurandy do Nascimento Silva, Daisy Jereissati Barbosa Lima, Paulo Michel Pinheiro Ferreira, Flavia Pereira da Silva Airoldi, Cláudia Pessoa, Antonia Maria das Graças Lopes Citó

**Affiliations:** 1 Department of Chemistry, Federal University of Piauí, Teresina 64049-550, PI, Brazil; E-Mails: nayaninhanery@hotmail.com (N.B.N.M.); bquirinoa@hotmail.com (B.Q.A.); 2 Postgraduate Program in Pharmaceutical Sciences, Federal University of Piauí, Teresina 64049-550, PI, Brazil; E-Mails: jurandy@ifpi.edu.br (J.N.S.); pmifepe@yahoo.com.br (P.M.P.F.); 3 Department of Physiology and Pharmacology, Federal University of Ceará, Fortaleza 60430-270, CE, Brazil; E-Mails: daisylima@gmail.com (D.J.B.L.); cpessoa@ufc.br (C.P.); 4 Department of Biophysics and Physiology, Federal University of Piauí, Teresina 64049-550, PI, Brazil; 5 Laboratory of Organic Geochemistry, University of Campinas, São Paulo 13083-970, SP, Brazil; E-Mail: flavia_aguayo@yahoo.com.br; 6 Oswald Cruz Foundation, Fortaleza 60180-900, CE, Brazil

**Keywords:** *Mimosa caesalpiniifolia*, phenolic compounds, cytotoxicity, betulinic acid

## Abstract

*Mimosa caesalpiniifolia* is a native plant of the Brazilian northeast, and few studies have investigated its chemical composition and biological significance. This work describes the identification of the first chemical constituents in the ethanolic extract and fractions of *M. caesalpiniifolia* stem bark based on NMR, GC-qMS and HRMS analyses, as well as an assessment of their cytotoxic activity. GC-qMS analysis showed fatty acid derivatives, triterpenes and steroid substances and confirmed the identity of the chemical compounds isolated from the hexane fraction. Metabolite biodiversity in *M. caesalpiniifolia* stem bark revealed the differentiated accumulation of pentacyclic triterpenic acids, with a high content of betulinic acid and minor amounts of 3-oxo and 3β-acetoxy derivatives. Bioactive analysis based on total phenolic and flavonoid content showed a high amount of these compounds in the ethanolic extract, and ESI-(−)-LTQ-Orbitrap-MS identified caffeoyl hexose at high intensity, as well as the presence of phenolic acids and flavonoids. Furthermore, the evaluation of the ethanolic extract and fractions, including betulinic acid, against colon (HCT-116), ovarian (OVCAR-8) and glioblastoma (SF-295) tumour cell lines showed that the crude extract, hexane and dichloromethane fractions possessed moderate to high inhibitory activity, which may be related to the abundance of betulinic acid. The phytochemical and biological study of *M. caesalpiniifolia* stem bark thus revealed a new alternative source of antitumour compounds, possibly made effective by the presence of betulinic acid and by chemical co-synergism with other compounds.

## 1. Introduction

*Mimosa* L. (Fabaceae) is the second largest genus of the Mimosoideae subfamily and comprises approximately 530 species, distributed mainly in South and Central America [1,2,3]. Despite the great biodiversity of the genus *Mimosa*, phytochemical and pharmacological studies are restricted to about twenty representatives, of which *Mimosa pudica* and *Mimosa tenuiflora* are the most commonly investigated species.

*Mimosa* species are rich in polyphenol compounds such as flavonoids (e.g., flavones, flavonols), lignans and other phytochemicals, including alkaloids, terpenoids, steroids and saponins [4,5,6,7,8]. In pharmacological investigations, *Mimosa* species are mainly characterised by their antioxidant potential [4,9] and antimicrobial activity [10,11]. However, studies investigating acetylcholinesterase [12], antiulcerogenic [13], antidiabetic [14], anti-inflammatory [15], antifungal [16], antinociceptive [17] and cytotoxic effects [18] of *Mimosa* species have also been described.

*Mimosa caesalpiniifolia* Benth. (syn. *Mimosa caesalpiniaefolia*) is a native plant to northeastern Brazil, popularly known as “unha-de-gato”, “sabiá”, “angiquinho-sabiá” and “sansão do campo” [19,20]. Due to their therapeutic effects, the stem bark and flowers have been used in traditional medicine for the treatment of bronchitis, skin infections and injuries [21] and for inflammation and hypertension [22], respectively. Therefore, detailed phytochemical studies are necessary to identify the active compound(s) for phytotherapeutic product development and to possibly aid in the search for new drugs. Taking into account previous chemical and pharmacological studies of *M. caesalpiniifolia* [23,24] and screenings for the antiproliferative activity of Brazilian species, this work describes the first phytochemical study of *M. caesalpiniifolia* stem bark. Non-polar compounds and acid derivatives of hexane and dichloromethane fractions were analysed by GC-qMS, and polar compounds from ethanolic extract were identified by HRMS and MS^n^ experiments. Additionally, ethanolic extract, fractions and isolated compounds were evaluated for cytotoxic activity against the HCT-116, OVCAR-8 and SF-295 human tumour cell lines.

## 2. Results and Discussion

The extraction of *M. caesalpiniifolia* stem bark yielded a crude ethanolic extract with 4.2% (w/w) extractable matter. Partitioning of the EtOH extract of *M. caesalpiniifolia* with different solvents yielded fractions at extraction efficiencies ranging from 7.4% to 37.7%. The aqueous fraction had the highest extraction efficiency (37.7%, w/w), whereas the *n*-hexane fraction presented the lowest extraction efficiency (7.4%, w/w). The efficiency of fractionation for different fractions in descending order was as follows: aqueous > dichloromethane > ethyl acetate > hexane.

### 2.1. Phytochemical Analysis

**Figure 1:**
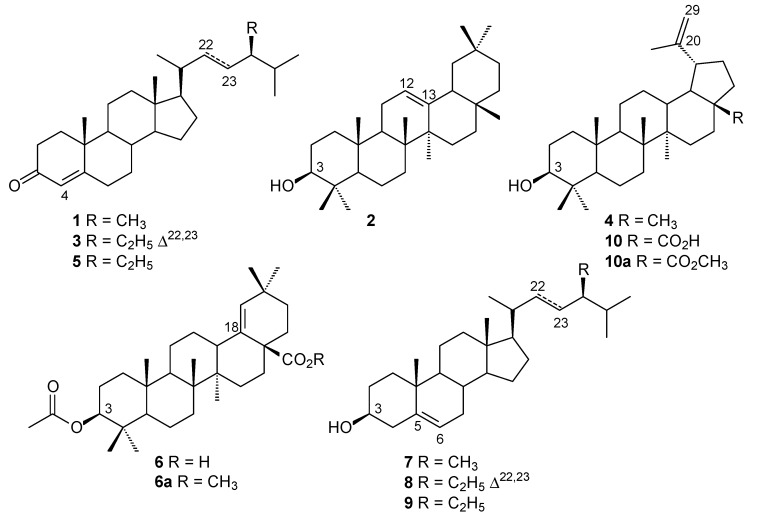
Chemical constituents isolated from the hexane fraction of the ethanolic stem bark extract of *Mimosa caesalpiniifolia*.

The chromatographic separation of the hexane fraction of ethanolic stem bark extract from *Mimosa caesalpiniifolia* led to the isolation and characterisation of campestenone (**1**), β-amyrin (**2**), stigmasta-4,22-dien-3-one (**3**), lupeol (**4**), sitostenone (**5**), 3β-acetoxy-olean-18-en-28-oic acid (**6**), campesterol (**7**), stigmasterol (**8**), sitosterol (**9**) and betulinic acid (**10**) (Figure 1). The structures **1**–**10** were confirmed by comparison of TLC, GC-qMS, HRAPCIMS, ^1^H-, ^13^C-NMR and DEPT analyses with data from the literature [25,26,27].

The ^1^H- and ^13^C-NMR spectra of subfraction F2 showed signals at δ_H_ 3.19 (*dd*, *J =* 5.1 and 11.2 Hz, H-3) and δ_C_ 79.2 characteristic of 3β-OH triterpenoids. The signals at δ_H_ 4.56 and 4.68 (H29α and H29β) and δ_C_ 109.5 (C-29) and 151.1 (C-20) were assigned to olefinic carbons in the lup-20(29)-ene skeleton. GC-qMS analysis showed a peak of high relative abundance (60.9%) with a molecular ion at *m/z* 426 [M^+•^], which suggested lupeol (**4**). Furthermore, the signal at δ_H_ 5.73, attributed to olefinic hydrogen H-4 in a steroidal nucleus with a carbonyl group at C-3, and the signal at δ_C_ 199.9 (C-3) suggested a 3-ketosteroid structure. The presence of stigmasta-4,22-dien-3-one (**3**) was suggested by signals at δ_H_ 5.02 and 5.15 (*dd*, *J =* 8.6 and 15.2 Hz), assigned to methine protons H-22 and H-23; signals at δ_C_ 129.6 (C-22) and 138.2 (C-23) characteristic of stenone-like compounds; and the results of GC-qMS analysis, which showed a peak with 11.4% relative abundance at *m/z* 410 ([M^+•^], EIMS data) compatible with formula C_29_H_46_O.

Other peaks in the total ion chromatogram (TIC) were identified as campestenone + β-amyrin (**1** + **2**, 8.7%), sitostenone (**5**, 7.5%) and 3β-*O*-acetyl-olean-18-en-28-oic acid methyl ester (**6a**, 11.5%) (Figure S1, Supplementary Materials). The triterpene β-amyrin was detected in the subfraction F2 in coelution with campestenone, based on the extracted ion chromatogram (EIC) results at *m/z* 124 (3-ketosteroid) and *m/z* 218 (base peak, 3β-hydroxy-olean-12-ene skeleton) and GC-qMS analysis of the subfraction F2-1. This subfraction showed a pentacyclic triterpene mixture of β-amyrin and lupeol (**2** + **4**) with relative abundance at 3.0% and 97.0%, respectively, based on retention time and mass spectral data (Figure S1, Supplementary Materials). The triterpenoid 3β-acetoxy-olean-18-en-28-oic acid methyl ester (**6a**) was only detected in the GC-qMS analysis of methylated subfraction F2, *i.e.*, the chemical compound **6a** occurs in *M. caesalpiniifolia* stem bark as free triterpenic acid **6**.

The steroid mixture (F3, **7** + **8** + **9**) showed a multiplet at δ_H_ 3.52 (H-3) and δ_H_ 5.34 (*d*, 5.2 Hz), assigned to H-6 for campesterol (**7**), stigmasterol (**8**) and sitosterol (**9**), and at δ_H_ 5.01 and 5.15 (*dd*, *J* = 8.6 and 15.2 Hz), assigned to methine protons H-22 and H-23 for stigmasterol (**8**). The ^13^C-NMR spectra showed oxymethine carbon at δ_C_ 71.9 and δ_C_ 140.9 (C-5) and 121.8 (C-6) for steroid 3β-OH with a ∆^5^ skeleton (**7** + **8** + **9**). The methine carbons at δ_C_ 129.4 (C-23) and 138.4 (C-22) confirmed a stigmasterol compound (**8**). The assignment of steroid side-chain signals in ^13^C-NMR analysis allowed the identification of campesterol (**7**) and sitosterol (**9**), based on signals at δ_C_ 30.4, 39.0, 32.6, 20.4, 18.4 and 15.5; and δ_C_ 26.2, 46.0, 29.3, 20.0, 19.2 and 23.2, respectively, attributed to C-23, C-24, C-25, C-26, C-27 and C-28 in the steroids [27]. The steroid mixture (subfraction F3) showed a TIC in GC-qMS analysis (Figure S1, Supplementary Materials) with three peaks, corresponding to the following molecular ions: *m/z* 400 (**7**, 10.1%), 412 (**8**, 72.1%) and 414 (**9**, 17.8%), respectively; stigmasterol (**8**) was identified as a major compound.

The ^1^H- and ^13^C-NMR analysis of subfraction F4 showed signals similar to a 3β-hydroxy-lup-20(29)-ene skeleton, except for the presence of a signal at δ_C_ 181.4, which indicated a carbonyl group in C-28 and the absence of δ_C_ 18.1 (CH_3_). The total ion chromatogram showed one peak for the methylated sample (Figure S1, Supplementary Materials) and EIMS revealed an ion at *m/z* 470 [M^+•^] in GC-qMS analysis and fragment ions at *m/z* 189 (base peak, 100%) and *m/z* 207 (37%). This result suggests triterpenoid structures characteristic of a lupane skeleton, and the ion at *m/z* 262 (27%) is indicative of a triterpenoid C-28 methyl ester derivatives. HRAPCIMS in negative mode showed a deprotonated ion at *m/z* 455.3510 [M−H]^−^ (calculated for C_30_H_47_O_3_, 455.3524) and 469.3672 [M−H]^−^ (calculated for C_31_H_49_O_3_, 469.3681), which confirmed the structures of betulinic acid (**10**) and methyl betulinate (**10a**, a synthetic derivative), respectively.

#### 2.1.1. Non-Polar Compounds and Acid Derivatives of *M. caesalpiniifolia* by GC-qMS

**Figure 2:**
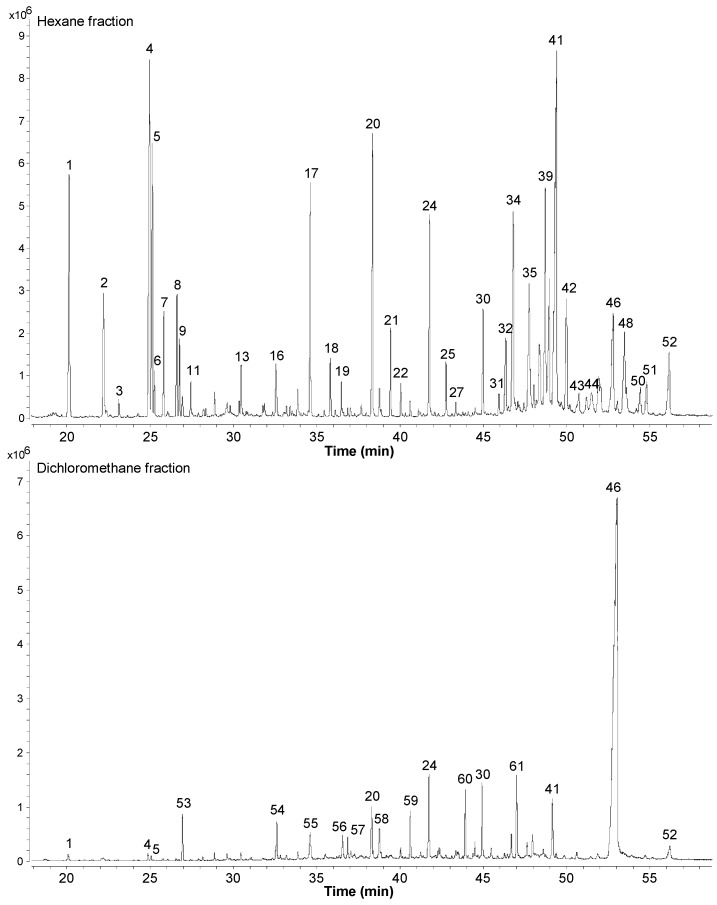
Total ion chromatogram (TIC) of hexane and dichloromethane fractions of *Mimosa caesalpiniifolia* in GC-qMS analysis.

Analysis of the chemical composition of extracts, fractions and natural compounds by GC-qMS is a useful tool in phytochemical studies for the separation and identification of individual compounds from organic matrices, especially in bioactivity-guided studies [28]. To determine the phytoconstituents and fatty acids of *M. caesalpiniifolia* stem bark, the hexane and dichloromethane fractions were methylated using diazomethane solution. The GC-qMS profiles of the methylated hexane and dichloromethane fractions from the ethanolic extract of *M. caesalpiniifolia* are shown in Figure 2.

The relative abundance (%), molecular ion, formula and retention time of the compounds identified in the hexane and dichloromethane fractions of *M. caesalpiniifolia* by GC-qMS analysis are shown in Table 1.

**Table 1:** Chemical compounds (%) identified by GC-qMS analysis of hexane and dichloromethane fractions of ethanolic stem bark extract of *Mimosa caesalpiniifolia*.

Chemical Constituents		Molecular Formula	EIMS [M^+•^]	Rt (min)	HEX (%)	DCM (%)	
1	Methyl hexadecanoate C16:0	C_17_H_34_O_2_	270	20.16	6.05	0.33	
2	Ethyl hexadecanoate C16:0	C_18_H_36_O_2_	284	22.23	2.43		
3	Methyl heptadecanoate C17:0	C_18_H_36_O_2_	284	23.14	0.34		
4	Methyl (9*Z*,12*Z*) octadeca-9,12-dienoate C18:2	C_19_H_34_O_2_	294	24.98	8.05	0.32	
5	Methyl (9*Z*)-octadec-9-enoate C18:1	C_19_H_36_O_2_	296	25.15	5.13	0.13	
6	Methyl (11*Z*)-octadec-11-enoate C18:1	C_19_H_36_O_2_	296	25.27	0.62		
7	Methyl octadecanoate C18:0	C_19_H_38_O_2_	298	25.84	1.73		
8	Ethyl (9*Z*,12*Z*)-octadeca-9,12-dienoate C18:2	C_20_H_36_O_2_	308	26.63	2.04		
9	Ethyl (9*Z*)-octadec-9-enoate C18:1	C_20_H_38_O_2_	310	26.78	1.21		
10	Ethyl(11*Z*)-octadec-11-enoate C18:1	C_20_H_38_O_2_	310	26.94	0.44		
53	Unidentified			26.97		2.17	
11	Ethyl octadecanoate C18:0	C_20_H_40_O_2_	312	27.46	0.53		
12	Methyl (10*Z*,13*Z*)-nonadeca-10,13-dienoate C19:2	C_20_H_36_O_2_	308	27.92	0.08		
13	Methyl nonadecanoate C19:0	C_20_H_40_O_2_	312	28.23	0.17		
14	Methyl eicosanoate C20:0	C_21_H_42_O_2_	326	30.49	0.75		
15	Ethyl eicosanoate C20:0	C_22_H_44_O_2_	340	31.89	0.14		
16	Methyl heneicosanoate C21:0	C_22_H_44_O_2_	340	32.56	1.14		
54	Unidentified			32.62		1.32	
17	Methyl docosanoate C22:0	C_23_H_46_O_2_	354	34.63	3.85		
55	Unidentified			34.64		1.76	
18	Ethyl docosanoate C22:0	C_24_H_48_O_2_	368	35.84	0.84		
19	Methyl tricosanoate C23:0	C_24_H_48_O_2_	368	36.49	0.49		
56	Unidentified			36.56		0.79
57	Unidentified			36.87		0.76
20	Methyl tetracosanoate C24:0	C_25_H_50_O_2_	382	38.36	4.95	2.16
58	Unidentified			38.78		1.21
21	Ethyl tetracosanoate C24:0	C_26_H_52_O_2_	396	39.45	1.29	
22	Methyl pentacosanoate C25:0	C_26_H_52_O_2_	396	40.06	0.49	
59	Unidentified			40.62		1.73
23	Ethyl pentacosanoate C25:0	C_27_H_54_O_2_	410	41.14	0.1	
24	Methyl hexacosanoate C26:0	C_27_H_54_O_2_	410	41.78	3.29	3.08
25	Ethyl hexacosanoate C26:0	C_28_H_58_O_2_	424	42.78	0.74	
26	Stigmastatriene	C_29_H_46_	394	43.13	0.04	
27	Methyl heptacosanoate C27:0	C_28_H_56_O_2_	424	43.37	0.22	
60	Unidentified			43.93		2.82
28	Stigmastadiene	C_29_H_48_	396	44.10	0.06	
29	α-Tocopherol (vitamin E)	C_29_H_50_O_2_	430	44.83	0.02	
30	Methyl octacosanoate C28:0	C_29_H_58_O_2_	438	44.99	1.88	3.19
31	Ethyl octacosanoate C28:0	C_30_H_60_O_2_	452	45.95	0.36	
32	Campesterol	C_28_H_48_O	400	46.36	1.67	
33	Campestanol	C_28_H_50_O	402	46.52	0.07	
34	Stigmasterol	C_29_H_48_O	412	46.81	4.75	
61	Unidentified			47.01		3.47
35	Sitosterol	C_29_H_50_O	414	47.76	2.97	
36	β-Amyrenone	C_30_H_48_O	424	47.81	0.4	
37	β-Amyrin	C_30_H_50_O	426	48.36	1.05	
38	Campestenone	C_28_H_46_O	398	48.41	0.87	
39	Lupenone	C_30_H_48_O	424	48.74	5.35	
40	Stigmasta-4,22-dien-3-one	C_29_H_46_O	410	48.97	2.47	
41	Lupeol	C_30_H_50_O	426	49.42	14.7	3.26
42	Sitostenone	C_29_H_48_O	412	50.01	2.74	
43	3-Oxo-olean-18-en-28-oic acid methyl ester	C_31_H_48_O_3_	468	50.75	0.56	
44	3-Oxo-olean-12-en-28-oic acid methyl ester	C_31_H_48_O_3_	468	51.54	0.26	
45	3-Oxo-lup-20(29)-en-28-oic acid methyl ester	C_31_H_48_O_3_	468	51.91	1.20	
46	3β-Hydroxy-lup-20(29)-en-28-oic acid methyl ester	C_31_H_50_O_3_	470	52.81	4.25	70.3
47	3-Oxo-urs-12-en-28-oic acid methyl ester	C_31_H_48_O_3_	468	53.05	0.24	
48	3β-Acetoxy-olean-18-en-28-oic acid methyl ester	C_33_H_52_O_4_	512	53.49	2.76	
49	Stigmastane-3,6-dione	C_29_H_48_O_2_	428	53.62	0.36	
50	3β-Acetoxy-olean-12-en-28-oic acid methyl ester	C_33_H_52_O_4_	512	54.43	0.71	
51	3β-Acetoxy-lup-20(29)-en-28-oic acid methyl ester	C_33_H_52_O_4_	512	54.80	0.99	
52	3β-Acetoxy-urs-12-en-28-oic acid methyl ester	C_33_H_52_O_4_	512	56.16	2.20	1.24

Compound identification in the GC-qMS analysis was performed using retention time and interpretation of the mass spectra (molecular ion [M^+•^], base peak and main fragments) in comparison with mass spectra of isolated compounds, computational libraries and literature data [29,30,31,32].

Major components identified in the hexane fraction of *M. caesalpiniifolia* were fatty acid derivatives (49.35%), triterpenes (34.67%) and steroids (16.00%), and the dichloromethane fraction was dominated by triterpenes (74.80%). The total fatty acid composition of the hexane fraction showed methyl ester (*m/z* 74) and ethyl ester (*m/z* 88), predominantly, with fatty acid methyl esters (39.23%), specially methyl hexadecanoate (6.05%), methyl tetracosanoate (4.95%) and unsaturated fatty acid methyl ester C18:2 *cis*-9,*cis*-12 (8.05%) and C18:1 *cis*-9 (5.13%). Fatty acid ethyl esters occur naturally. There is evidence for the presence of ethyl ester C16:0 to C26:0 in the non-methylated subfraction F1 of the *n*-hexane fraction on a silica gel column (Section 3.3).

Steroid compounds identified in the hexane fraction of *M. caesalpiniifolia* showed a similar distribution pattern to that observed in *M. artemisiana*, including the presence of stenols and stenones [6]. The steroids identified were subdivided into four classes: non-oxygenated steroids (0.10%), stenols (9.46%), stenones (6.08%) and diketosteroids (0.36%). The major steroids (>2.0%) were stigmasterol (4.75%), sitosterol (2.97%), sitostenone (2.74%) and stigmasta-4,22-dien-3-one (2.47%). In the literature, stigmasta-4,22-dien-3-one has shown moderate antitumoural activity [25].

Triterpene profiles in the hexane and dichloromethane fractions showed a variation of 34.67% and 74.80%, respectively. The main compounds in the hexane and dichloromethane fractions were lupeol (14.70%) and betulinic acid (70.30%), respectively. In these fractions, 3-ketotriterpenoids and derivatives were observed. The identification of triterpenes was performed based on mass spectra (EIMS) and characteristic fragments; the 3β-hydroxy-olean-12-ene and 3β-hydroxy-lup-20(29)-ene skeletons showed base peaks at *m/z* 218 and 189, respectively, and 3-ketotriterpenoids showed *m/z* 205 [29,30].

In addition, the presence of triterpenic acid derivatives (13.17% to 71.54%) indicated the great metabolic biodiversity of *M. caesalpiniifolia*, mostly the high accumulation of betulinic acid (3β-hydroxy-lup-20(29)-en-28-oic acid) in the stem bark. The triterpenic acid derivatives showed *m/z* 262 for the acid methyl ester group at C-28, except in olean-18-ene acid methyl ester, which showed *m/z* 248. In the urs-12-ene acid methyl ester skeleton, an additional fragment at *m/z* 133 (base peak) was observed [29,30].

On the other hand, α-tocopherol was the single phenolic compound detected in the hexane fraction (relative intensity 0.02%) using the GC-qMS extracted ion chromatogram (EIC), at *m/z* 165. The fragment at *m/z* 165 [C_10_H_13_O_2_]^+^ resulted from hydrogen rearrangement and retro-Diels-Alder cleavage of the pyran ring (Figures S2 and S3, Supplementary Materials). In comparative chromatograms, α-tocopherol showed a relative abundance on the order of 100 times less than methyl octacosanoate.

The presence of phenolic compounds in the dichloromethane fraction was detected using EIC *m/z* 77, 91 and 105, which correspond to related benzene ring compounds, and *m/z* 94 and 108 for phenols, including a base peak at *m/z* 272 [M^+•^] indicative of C_6_-C_3_-C_6_ derivatives, in accordance with the NIST mass library. However, phenolic compounds in the dichloromethane fraction were found at less than 0.05% (trace level), which complicates their identification due to poor-quality spectra.

#### 2.1.2. Total Phenolic and Flavonoid Contents of *M. caesalpiniifolia* Stem Bark

The stem bark of *M. caesalpiniifolia* is characterised by the common occurrence of polyphenols and tannins (water-soluble phenolic compounds) [33]. The importance of polyphenols and related compounds in *Mimosa* species is associated with biological and pharmacological properties, such as antioxidant, cytotoxic and antimicrobial activity. Phytochemical investigation of *Mimosa invisa* showed the presence of phenolic substances, which include flavonoids, flavonoid glycosides and lignans with promising antiradical activity (e.g., quercetin, a well-known natural antioxidant) [5]. Two phenolic compounds (a deoxyflavone derivative and flavolignan) isolated from *Mimosa diplotricha* were active in an antiproliferative assay against tumour cell lines [4]. The cinnamic acid diterpenyl ester isolated from *Mimosa pudica* was highly active against the microorganisms *Malassezia pachydermatis*, *Candida albicans* and *Staphylococcus aureus* [34].

The total phenolic content (TPC) and total flavonoid content (TFC) in the ethanol extract and fractions of *M. caesalpiniifolia* are shown in Table 2. TPC values are expressed in milligrams of gallic acid equivalent per gram of dried plant material (mg GAE/g DPM), determined by the Folin-Ciocalteu method using gallic acid as a standard. TFC values are expressed in milligrams of rutin equivalent per gram of dried plant material (mg RE/g DPM), determined by the aluminium complex method using a rutin analytical curve.

**Table 2:** Total phenolic and flavonoid contents in ethanolic extract and fractions of *Mimosa caesalpiniifolia* stem bark.

Samples	TPC mg GAE/g DPM	TFC mg RE/g DPM
Ethanolic extract	14.80 ± 0.30	1.81 ± 0.14
Hexane fraction	0.16 ± 0.02	-
Dichloromethane fraction	0.56 ± 0.01	0.35 ± 0.01
Ethyl acetate fraction	3.17 ± 0.04	0.34 ± 0.02
Aqueous fraction	6.42 ± 0.09	0.68 ± 0.03

Notes: Mean ± standard deviation; Total phenolic content (TPC); Total flavonoid content (TFC); milligrams of gallic acid equivalent per gram of dry plant material (mg GAE/g DPM); milligrams of rutin equivalent per gram of dry plant material (mg RE/g DPM).

The ethanolic extract had the highest total phenolic content (14.8 mg GAE/g DPM), followed by the aqueous fraction (6.42 mg GAE/g DPM), whereas the hexane fraction had the lowest content (0.16 mg GAE/g DPM). It was observed that the phenolic content of the extract and fractions of *M. caesalpiniifolia* obeyed a decreasing order: ethanol > aqueous > ethyl acetate > dichloromethane > hexane. Phenolic compounds in the hexane fraction were confirmed by GC-qMS analysis, which showed a lower accumulation of α-tocopherol in *M. caesalpiniifolia* stem bark (Section 2.1.1.).

In the present investigation, the total flavonoid content (TFC) ranged from 0.34 to 1.81 mg RE/g DPM. The ethanol extract had the highest TFC (1.81 mg RE/g DPM), followed by the aqueous extract with 0.68 mg RE/g DPM. TFC extracted in solvents with different polarity were found to obey the following decreasing order: ethanol > aqueous > dichloromethane ≥ ethyl acetate (*p* < 0.05, one-way ANOVA). Generally, flavonoids are rarely found in extractions and partitioning with low-polarity solvents such as *n*-hexane [35]. According to GC-qMS analysis, flavonoids and C_6_-C_3_-C_6_ derivatives were not detected in the hexane fraction. Therefore, phenolic acids, polyphenol flavonoid-like compounds and tannins in the ethanolic extract of *M. caesalpiniifolia* stem bark were investigated by ESI(−)-MS.

#### 2.1.3. Identification of Phenolic Compounds in *M. caesalpiniifolia* by ESI(−)-LTQ-Orbitrap-MS

**Figure 3:**
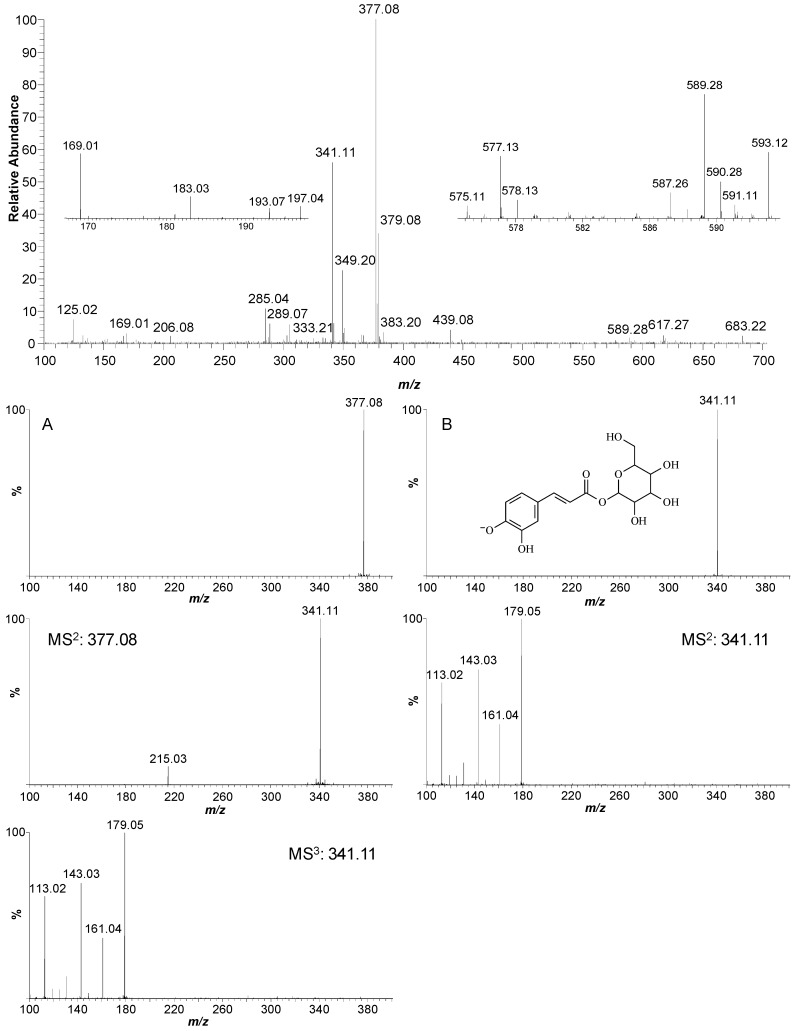
High-resolution fingerprint profile of ethanolic stem bark extract of *Mimosa caesalpiniifolia*. (**A**) MS^n^ spectra of caffeoyl hexoside derivative (*m/z* 377.08 [M−H]^−^) and (**B**) MS^n^ spectra of caffeoyl hexoside (*m/z* 341.11 [M−H]^−^).

In this study, several phenolic compounds were tentatively identified in the ethanolic extract of *M. caesalpiniifolia* stem bark using negative electrospray ionisation coupled to a linear ion trap-orbitrap hybrid mass spectrometer (ESI(−)-LTQ-Orbitrap-MS) in the scan mode and multi-stage mass analysis (MS^n^) (Figure 3). This method provides molecular and structural information for chemical identification. The phenolic composition of ethanolic extract was analysed because this extract had the highest concentration of total phenols and flavonoids, 14.8 mg GAE/g DPM and 1.81 mg RE/g DPM (Table 2), respectively.

The ESI(−)-MS signal at *m/z* 125.02 [M−H]^−^ suggested a trihydroxybenzene derivative, possibly pyrogallol. Hydroxybenzoic acids, with a C_6_-C_1_ chemical structure, showed MS^2^ fragments with a loss of CO_2_ [M−H−44]^−^. The [M−H]^−^ ion at *m/z* 169.01 was characterised as gallic acid, and the signals at *m/z* 183.03 and 197.04 were identified as gallic acid derivatives: methyl gallate and ethyl gallate, respectively. Gallic acid and derivatives have been reported in *Mimosa* species [36]. The ion at *m/z* 193.07 [M−H]^−^ was identified as ferulic acid, a C_6_-C_3_ structure. The deprotonated ion at *m/z* 206.08 was assigned to a possible nitrogen-containing compound based on the odd-mass molecular ion.

The [M−H]^−^ ion at *m/z* 285.04 with an MS^2^ fragment ion at *m/z* 241.05 [M−CO_2_−H]^−^ and some typical fragments of dihydroxybenzoic acid at *m/z* 153.02 [M−132−H]^−^ and 109.32 [M−132−CO_2_−H]^−^, following the loss of a pentose sugar unit (132 Da) and decarboxylation, possibly correspond to gentisic acid aglycone (2,5-dihydroxybenzoic acid) and hydroquinone, respectively. Therefore, the ion at *m/z* 285.04 [M−H]^−^ was identified as gentisic acid pentoside, according to previous reports that analysed the *Mimosa* genus [36].

The [M−H]^−^ ion at *m/z* 289.07 (C_15_H_14_O_6_) showed a MS^2^ fragment ion at *m/z* 245.08 [M−CH_2_CHOH−H]^−^ and was identified as catechin, a procyanidin monomer. Two types of procyanidin dimer were found in *M. caesalpiniifolia* stem bark. The low signal intensity of the [M−H]^−^ion at *m/z* 575.11 indicated a low accumulation level of A-type procyanidin dimers (C_30_H_24_O_12_), whereas the [M−H]^−^ ion at *m/z* 577.13 showed a high level of B-type procyanidin dimers (C_30_H_26_O_12_) [37]. Catechin and procyanidin dimers have previously been described in *M. caesalpiniifolia* leaves [24].

The [M−H]^−^ ion at 341.11 showed a MS^2^ ion at *m/z* 179.05 (caffeic acid), a loss of 162 Daltons (hexose moiety); fragment ions at *m/z* 161.04 [M−162−H_2_O−H]^−^, 143.03 [M−162−2H_2_O−H]^−^ and 113.02 [M−162−2H_2_O−H_2_CO−H]^−^, as well as a dimer ion at *m/z* 683.22 [2M−H]^−^, confirmed caffeoyl hexose (Figure 3A). The signal of a deprotonated molecular ion at *m/z* 377.08 [M−H]^−^ revealed MS^2^ fragment ions at *m/z* 341.11 [M−2H_2_O−H]^−^ and 215.03 [M−162−H]^−^, corresponding to the loss of a hexose unit (162 Da). The MS^3^ spectra for *m/z* 341.11 showed fragment ions at *m/z* 179.05, 161.04, 143.03 and 113.02, which confirmed a caffeoyl hexose derivative (Figure 3B), as previously described. The signal at *m/z* 377.08 possibly corresponds to an adduct water-bridged deprotonated molecular ion [M+2H_2_O−H]^−^ [38], which occurs due to the high concentration of caffeoyl hexose (*m/z* 341.11 [M−H]^−^) in the EtOH extract of *M. caesalpiniifolia*.

A deprotonated ion at m/z 593.12 [M−H]^−^ produced MS^2^ fragment ions at *m/z* 575.13 (base peak, [M−18]^−^), 561.12 [M−32]^−^, 547.10 [M−46]^−^, 473.10 [M−120]^−^, 447.08, 429.07, 411.07 and 285.04. According to the MS^n^ fragmentation data, *m/z* 593.12 [M−H]^−^ was confirmed to be vicenin-2 (C_27_H_30_O_15_) [39]. Despite the presence of various [M−H]^−^ ions in the ESI(−)-MS fingerprinting of ethanolic extract, the plant contained a high intensity of ions at *m/z* 377.08 and 341.11, which confirms a high content of caffeoyl hexose in *M. caesalpiniifolia* stem bark.

### 2.2. Cytotoxic Activity

**Figure 4:**
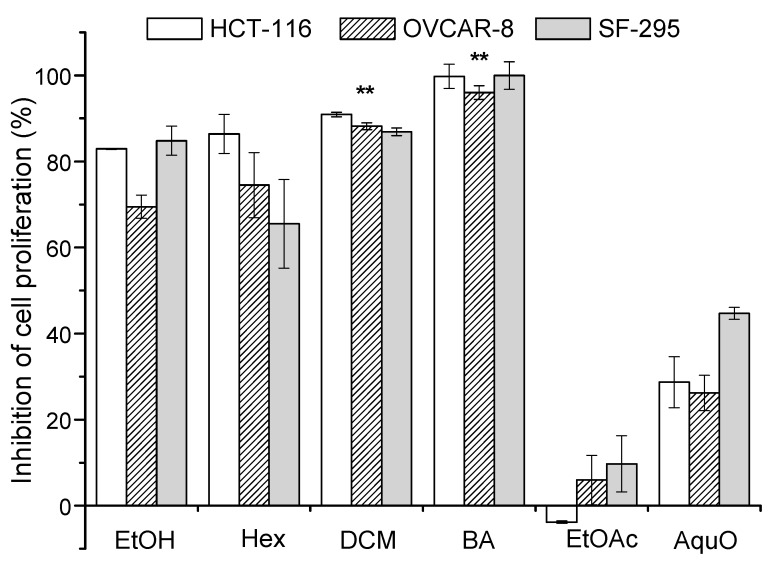
Percent inhibition of cell proliferation of extract and fractions of *Mimosa caesalpiniifolia* stem bark on cancer cell lines. HCT-116 (colon carcinoma), OVCAR-8 (ovarian carcinoma) and SF-295 (glioblastoma). EtOH (ethanolic extract), Hex (hexane fraction), DCM (dichloromethane fraction), BA (betulinic acid, isolated from hexane fraction), EtOAc (ethyl acetate fraction) and AquO (aqueous fraction). Asterisks (**) indicate no significant differences (*p* > 0.05, one-way ANOVA).

The ethanolic extract, partition fractions (hexane, dichloromethane, ethyl acetate and aqueous), betulinic acid and doxorubicin (positive control) were evaluated by MTT assay against colon (HCT-116), ovarian (OVCAR-8) and glioblastoma (SF-295) tumour cell lines. The positive control doxorubicin (at 0.3 µg/mL), a well-known anticancer drug that is widely used in clinical therapy [40], showed an inhibition of >83.0% for all tumour cell lines. The percentage inhibition of cell proliferation indicates that ethanolic extract and fractions showed moderate and high cytotoxic effects, with the exception of the ethyl acetate and aqueous fractions, as shown in Figure 4.

The results showed that the dichloromethane fraction and betulinic acid were the most active (>75.0%), with inhibition of cell proliferation above 86.5%, whereas the ethanolic extract and hexane fraction varied from 69.5% to 84.8% and 65.5% to 86.4%, respectively. These results demonstrated that the composition of the extract and fractions contains compounds that may contribute to chemical specificity and ability to interfere on the grown tumour cells, as observed in previous cytotoxicity studies of plant extracts and fractions [41].

As mentioned above, the effectiveness of inhibition is clearly affected by the chemical composition of the extract and fractions, which may be responsible for additive, synergetic, or antagonistic effects. Our results demonstrate that the hexane fraction showed decreased activity against the glioblastoma tumour cell strain (SF-295) when compared with ethanolic extract, with significantly different values (*p* < 0.05). In contrast, despite the low activity of the ethyl acetate and aqueous fractions, these fractions exhibited a strong growth inhibition effect in SF-295 tumour cells, when compared with the inhibition effects in the other cell strains. In the specific case of HCT-116 tumour cells, the negative percent inhibition observed for the ethyl acetate fraction showed that there was cell growth, *i.e.*, the fraction was inactive. The selectivity of the ethanolic extract and ethyl acetate and aqueous fractions against SF-295 tumour cells when compared with the hexane fraction is most likely due to the amount of polyphenol compounds, as shown in Table 2.

The ethanolic extract and hexane fraction showed no significant differences (one-way ANOVA, *p* > 0.05) for the inhibition of OVCAR-8 tumour cells. The one-way ANOVA test, applied to the data describing the inhibition of cell proliferation for the dichloromethane fraction and betulinic acid, showed that differences were not significant (*p* > 0.05) for all lines tested. In this context, the dichloromethane fraction and betulinic acid showed similarly powerful responses in the inhibition of the proliferation of neoplasic cells.

The cytotoxicity results might be related with the presence of betulinic acid, which was previously isolated from the hexane fraction (subfraction F4) and identified by GC-qMS in the dichloromethane fraction with a relative abundance of 70.30%. Literature, reports have shown the cytotoxic potential of betulinic acid [42,43]. Therefore, this study demonstrated that betulinic acid is the active compound responsible for the cytotoxic activity of *M. caesalpiniifolia* stem bark against tumour lines tested by the MTT assay.

It is important to highlight that the crude extract, which generally consists of a mixture of natural products such as phenolic acids, flavonoids, isoprenoids and primary metabolites such as fatty acid derivatives, was evaluated because it represents the most common approach in ethnopharmacological uses by Brazilian population. Moreover, it is likely that distinct bioactive molecules may jointly or independently contribute to the biological effects of plants [44,45]. Further studies are in progress to confirm these biological effects of *M. caesalpiniifolia* extracts.

## 3. Experimental Section

### 3.1. General

The solvents used in open column isolation, including *n*-hexane, dichloromethane, ethyl acetate and methanol, were analytical grade and were purchased from Synth (Labsynth, São Paulo, SP, Brazil). HPLC/spectro grade *n*-hexane, ethyl acetate and methanol were purchased from Tedia (Rio de Janeiro, RJ, Brazil). Water used was obtained from a Milli-Q system (18.2 MΩ cm, Milli Q-Plus system, Millipore Corporation, Bedford, MA, USA). Folin-Ciocalteu reagent was obtained from Merck (Darmstadt, Germany), aluminium chloride (AlCl_3_) was obtained from Fluka Analytical (Buchs, Switzerland) and rutin was purchased from Sigma Chemical Co. (St. Louis, MO, USA). Thin-layer chromatography (TLC) plates were prepared with silica gel 60 G (Sigma), sprayed with ceric sulphate solution and revealed on a hot plate at 100 °C. Atmospheric pressure column chromatography (CC) was performed on silica gel 60 (0.060–0.200 mm) from Acros Organics (Morris Plains, NJ, USA). NMR spectra were recorded on a INOVA spectrometer (Varian, Santa Clara, CA, USA) at 400 (^1^H) and 100 MHz (^13^C), using CDCl_3_ as solvent. Gas chromatography-quadrupole mass spectrometry (GC-qMS) analysis was performed on a GC7890A/VLMSD5975 system (Agilent Technologies Inc., Santa Clara, CA, USA) equipped with a DB-5 capillary column (J&W, 30 m × 250 mm × 0.25 μm) using NIST 0.8 and Wiley229 electron ionisation mass spectra (EIMS) computational libraries and authentic standards. High resolution mass spectra (HRMS) were obtained from a LTQ-Orbitrap XL (Thermo Fisher, Bremen, Germany) mass spectrometer equipped with ESI and APCI sources. Molecular absorbance measurements were determined using a Lambda 25 UV-vis spectrophotometer from PerkinElmer (Waltham, MA, USA). MTT (3-(4,5-dimethylthiazol-2-yl)-2,5-diphenyltetrazolium bromide) and doxorubicin (purity >98%) were purchased from Sigma Chemical Co. and RPMI-1640 medium was obtained from Cultilab (Campinas, SP, Brazil). Foetal bovine serum (FBS) and penicillin/streptomycin were purchased from Gibco-BRL (New York, NY, USA).

### 3.2. Plant Material

The stem bark of *M. caesalpiniifolia* was collected in Teresina, Piauí, Brazil in May 2010. The plant specimen was identified and deposited in the Graziela Barroso Herbarium with voucher specimen number TEBP 26,824.

### 3.3. Extraction and Isolation of Chemical Constituents

The stem bark was dried in air at room temperature and pulverised in a knife mill (MA680, Marconi Equipamentos Laboratório, Piracicaba, SP, Brazil). The stem bark powder (883.5 g) of *M. caesalpi niifolia* was extracted exhaustively with ethanol at 1:4 (w/v) plant material/solvent 10 consecutive times. The filtered and combined ethanolic extracts were concentrated under reduced pressure on a rotary evaporator (Laborota 4000, Heidolph Instruments, Schwabach, BY, Germany) and lyophilised (Edwards Micro Modulyo freeze dryer/Valpump VLP80 Savant, West Sussex, UK), yielding 36.7 g (4.2%) of dried EtOH extract. The stem bark ethanol extract (30.0 g) was suspended in MeOH-H_2_O (2:1, v/v) and subjected to successive partitioning, resulting in the following fractions: *n*-hexane (2.7 g, 7.4%), dichloromethane (3.9 g, 10.6%), EtOAc (3.7 g, 10.1%) and aqueous (13.8 g, 37.7%). The *n*-hexane fraction (1.0 g) was transferred to a silica gel chromatography column and eluted with *n*-hexane-EtOAc (v/v) using a gradient of increasing polarity as the mobile phase, and subfractions were combined based on their TLC profiles. Subfraction F1 (fraction 5, 90 mg, 9.0%), eluted with *n*-hexane-EtOAc (98:2, v/v), yielded a mixture dominated by fatty acid ethyl esters. GC-qMS analysis of the non-methylated subfraction F1 showed fatty acid ethyl esters with hydrocarbon chains C16:0 (27.54%), C18:2 (6.04%), C18:1 (6.28%), C18:0 (5.31%), C22:0 (8.70%), C24:0 (12.07%) and C26:0 (34.06%). Subfraction F2 (fractions 22 to 23, 142 mg, 14.2%) yielded a mixture of **1**–**6** with a predominance of lupeol (**4**). Silica gel column chromatography of subfraction F2 using isocratic *n*-hexane-EtOAc (8:2, v/v) yielded a mixture of β-amyrin (**2**) and lupeol (**4**) (F2-1, 43 mg). Subfraction F3 (fractions 28 to 30, 113 mg, 11.3%), eluted with *n*-hexane-EtOAc (9:1, v/v), was recrystallised from MeOH to give a mixture of campesterol (**7**), stigmasterol (**8**) and sitosterol (**9**). Subfraction F4 (fractions 34 to 37, 65 mg, 6.5%), eluted with *n*-hexane-EtOAc (8:2, v/v), was recrystallised from MeOH-DCM (3:1, v/v) to yield betulinic acid (**10**).

### 3.4. Analysis of Non-Polar Fractions of M. caesalpiniifolia by Gas Chromatography-Quadrupole Mass Spectrometry (GC-qMS)

The chemical constituents in the *n*-hexane and dichloromethane fractions were analysed by GC-qMS (GC7890A/VLMSD5975 system, Agilent Technologies) equipped with a DB-5 capillary column (J&W, 5% phenyl-95% methylpolysiloxane, 30 m × 250 mm × 0.25 μm). Samples were dissolved in *n*-hexane-EtOAc (1:1, v/v), after which previously prepared diazomethane diethyl ether solution was added. Sample aliquots of the solution (1 µL at 5 mg/mL w/v) were injected into a gas chromatograph in split mode (10:1). Helium was used as the carrier gas at a constant flow rate of 1 mL/min. The injector temperature was set to 310 °C. GC oven temperature program: initial temperature 150 °C (12 min); ramp at 4 °C/min to 290 °C (23 min). A mass spectrometer with quadrupole analyser was used, with electron ionisation (EI) at 70 eV, ion source at 300 °C, solvent delay time 8 min and mass range scan 40–650 Da. Chemical constituents were identified through the comparison of the obtained mass spectra with Wiley229 and NIST 0.8 computational libraries and authentic standards.

### 3.5. Determination and Identification of Polyphenols

#### 3.5.1. Total Phenol Content

Total phenol content (TPC) in the ethanol extract and fractions was measured using the Folin-Ciocalteu method described by Sousa *et al.* [46], with some modifications. Aliquots of dried samples dissolved in methanol (0.1 mL, 1000 μg/mL) were transferred to 10-mL volumetric flasks, followed by the addition of Folin-Ciocalteu reagent (0.5 mL) and distilled water (5 mL), and then samples were mixed for 1 min. Sodium carbonate (2 mL, 15% w/v) was added, and samples were stirred for 30 s. The volumetric flasks were filled to 10 mL with distilled water and incubated at room temperature for 2 h. The absorbance was measured at 750 nm using a UV-Vis spectrophotometer, and TPC content was determined in milligrams of gallic acid equivalent per gram of dry plant material (mg GAE/g DPM) using a gallic acid analytical curve (0.1–2.5 μg/mL, R = 0.999). All analyses were performed in triplicate (*n* = 3).

#### 3.5.2. Total Flavonoid Content

Total flavonoid content (TFC) in the ethanol extract and fractions was determined using the aluminium complex method and a rutin analytical curve as described by Ferreira *et al.* [34]. Aliquots of dried samples dissolved in methanol (0.3 mL, 1000 μg/mL) were transferred to 10 mL volumetric flasks, followed by the addition of acetic acid (0.24 mL), pyridine in methanol (4 mL, 20% v/v) and aluminium chloride methanolic solution (1 mL, 5% w/v). The volumetric flasks were filled to 10 mL with distilled water. These solutions were incubated at room temperature for 30 min, and the absorbance was measured at 420 nm using a UV-Vis spectrophotometer. TFC contents were expressed in milligrams of rutin equivalent per gram of dry plant material (mg RE/g DPM) using a rutin analytical curve (3.0–21.0 μg/mL, R = 0.999). All analyses were performed in triplicate (*n* = 3).

#### 3.5.3. Direct Infusion Mass Spectrometry Analysis of Ethanolic Extract of *M. caesalpiniifolia* Stem Bark by ESI(−)-LTQ-Orbitrap-MS

EtOH extract (20.0 mg) of *M. caesalpiniifolia* stem bark was submitted to solid-phase extraction using a C_18_ Strata cartridge (500.0 mg, Phenomenez, Torrance, CA, USA). The C_18_ Strata cartridge was activated with methanol (5 mL) and subsequently water (5 mL). Polyphenols were eluted with MeOH-H_2_O (5 mL, 8:2, v/v) in isocratic mode. The eluted fraction was evaporated under nitrogen flow, and the residue was reconstituted with 2 mL of MeOH-H_2_O (8:2, v/v) and filtered through a Millipore membrane filter (0.45 µm, PTFE) coupled to a syringe into a vial for ESI-(−)-LTQ-Orbitrap-MS analysis.

For polyphenol analysis, a LTQ Orbitrap mass spectrometer was equipped with an ESI source in negative ionisation mode. FTMS mass spectra were acquired at a resolution of 100,000 in the mass range *m/z* 100 to 1000 Da on the Orbitrap analyser. The MS full mode parameters were as follows: spray voltage 3.30 kV; sheath gas 8 (arbitrary units); capillary voltage −46 V; capillary temperature 300 °C; and tube lens −115.14 V. The multi-stage analysis (MS^n^) mode on the ion trap analyser for selected precursor ions was acquired by CID fragmentation using a normalised collision energy of 35.0. The data were processed using XCalibur software (version 2.1. Thermo Fischer Scientific Inc., San Jose, CA, USA), which provides possible elemental molecular formulas, accurate masses and isotopic patterns.

### 3.6. Cytotoxicity Assay

#### 3.6.1. Cell Culture

HCT-116 (colon); OVCAR-8 (ovarian) and SF-295 (glioblastoma) tumour cell lines (National Cancer Institute, Bethesda, MD, USA) were maintained in RPMI-1640 medium supplemented with 10% foetal bovine serum (FBS) and 1% antibiotics at 37 °C with a humidified atmosphere with 5% CO_2_ for cell growth.

#### 3.6.2. Evaluation of Cell Proliferation by MTT Assay

The cytotoxicity of ethanolic extract; the *n*-hexane, dichloromethane, ethyl acetate and aqueous fractions; and betulinic acid (isolated compound) and doxorubicin (positive control) was investigated by MTT assay [47], against HCT-116, OVCAR-8 and SF-295 cancer cells. This method analyses tumour cell growth by the ability of living cells to reduce the yellow dye 3-(4,5-dimethylthiazol-2-yl)-2,5-diphenyl-tetrazolium bromide (MTT) to a blue formazan product. Briefly, cells (0.1 × 10^6^ cells/well) were plated in 96-well plates and incubated for 72 h with a medium consisting of DMSO (control group); ethanol extract, fractions and subfractions at 50.0 μg/mL; or doxorubicin (0.3 μg/mL, positive control). Then, the supernatant was replaced by fresh medium containing 0.15 mL of MTT, and the cells were incubated for an additional 3 h. The plates were centrifuged, the formazan product was dissolved in DMSO and the absorbance was measured at 595 nm using a multiplate reader (DTX 880 Multimode Detector, Beckman Coulter Inc., Fullerton, CA, USA). The percentage inhibition of cell growth was calculated, according to Mahmoud *et al.* [41], using the software GraphPad Prism (GraphPad Software, Inc., La Jolla, CA, USA). The experiment was performed in triplicate, and the results were expressed as the mean ± standard deviation (SD).

### 3.7. Statistical Analysis

A statistical approach was designed and experimental data were evaluated using one-way analysis of variance (ANOVA) using the software Origin 8.0 (OriginLab Corporation, Northampton, MA, USA), with a significance level of *p* < 0.05.

## Supplementary Materials

Supplementary materials can be accessed at: http://www.mdpi.com/1420-3049/20/03/4204/s1.
