# Supplementary material for: Assessing Chemical Constituents of Mimosa caesalpiniifolia Stem Bark: Possible Bioactive Components Accountable for the Cytotoxic Effect of M. caesalpiniifolia on Human Tumour Cell Lines
Source: Molecules. 2015 Mar 5;20(3):4204–24. doi: 10.3390/molecules20034204 (PMC6272184; doi:10.3390/molecules20034204)
Supplement: Supplementary file 1 [file molecules-20-04204-s001.pdf]

Supplementary Materials

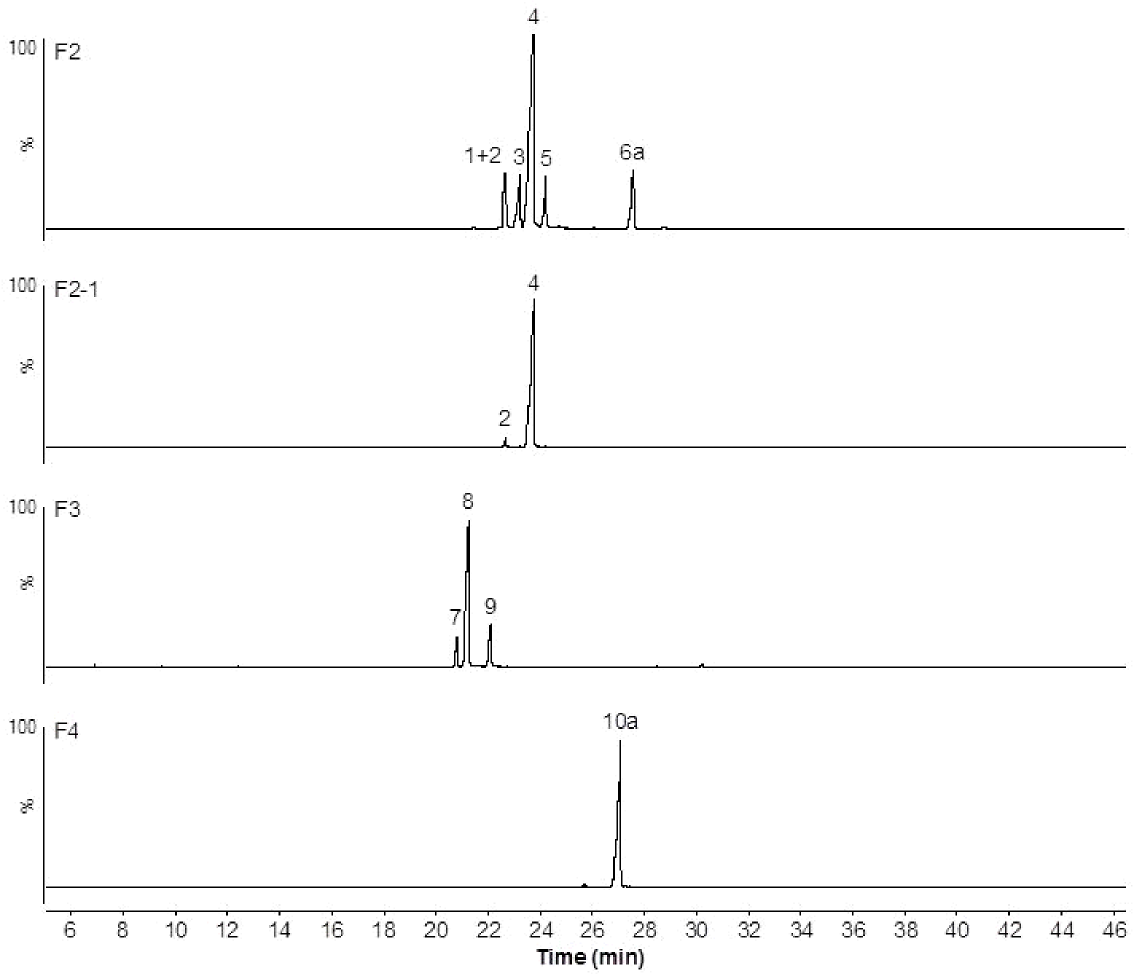

Figure S1. Total ion chromatogram (TIC) of the subfractions (F2, F2-1, F3 and F4) of silica gel CC hexane fraction of *Mimosa caesalpiniifolia* by GC-qMS analysis. F2: campestenone (**1**), β-amyrin (**2**), stigmasta-4,22-dien-3-one (**3**), lupeol (**4**), sitostenone (**5**), 3β-O-acetyl-olean-18-en-28-oic acid methyl ester (**6a**). F2-1: β-amyrin (**2**), lupeol (**4**). F3: campesterol (**7**), stigmasterol (**8**), sitosterol (**9**). F4: betulinic acid methyl ester (**10a**). *GC-qMS analysis - GC oven temperature: 200 °C (4 min) at 6 °C min^{-1} to 290 °C (15 min) at 2 °C min^{-1} to 305 °C (5 min) and solvent delay time: 5 min.

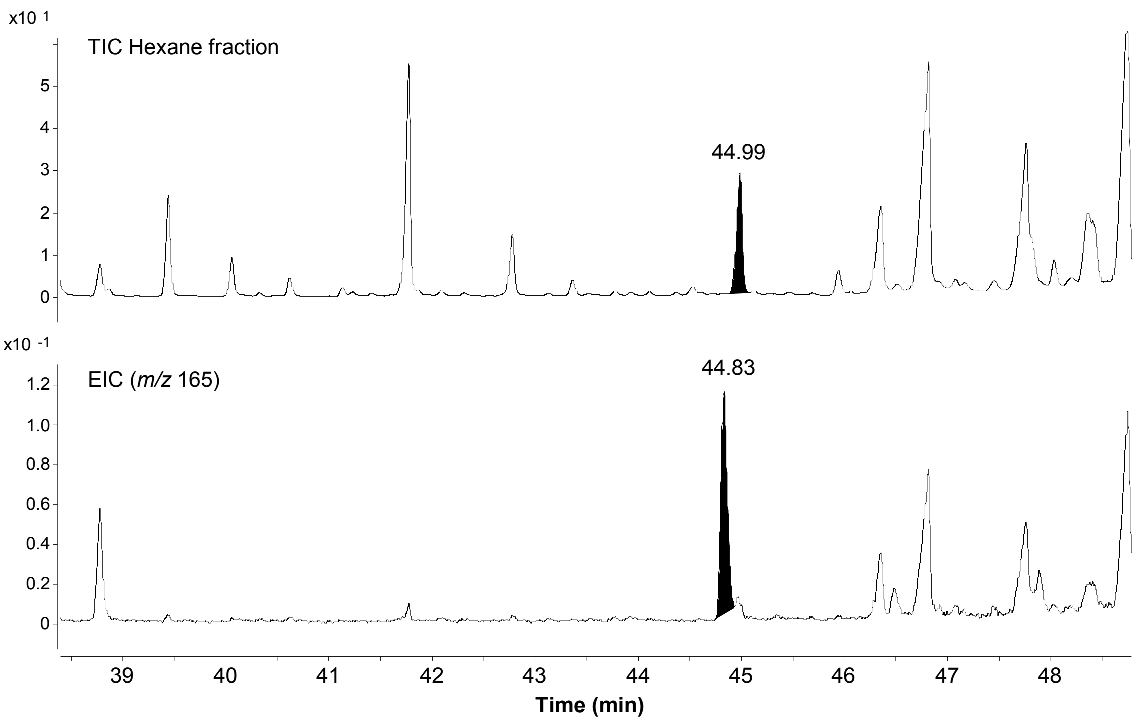

Figure S2. Total ion chromatogram (TIC) and extracted ion chromatogram (EIC) *m/z* 165 of hexane fraction from ethanolic stem bark extract of *Mimosa caesalpiniifolia*. Peak at 44.99 min: methyl octacosanoate ([$M^{+\bullet}$]: 438). Peak at 44.83 min: α-tocopherol ([$M^{+\bullet}$]: 430).

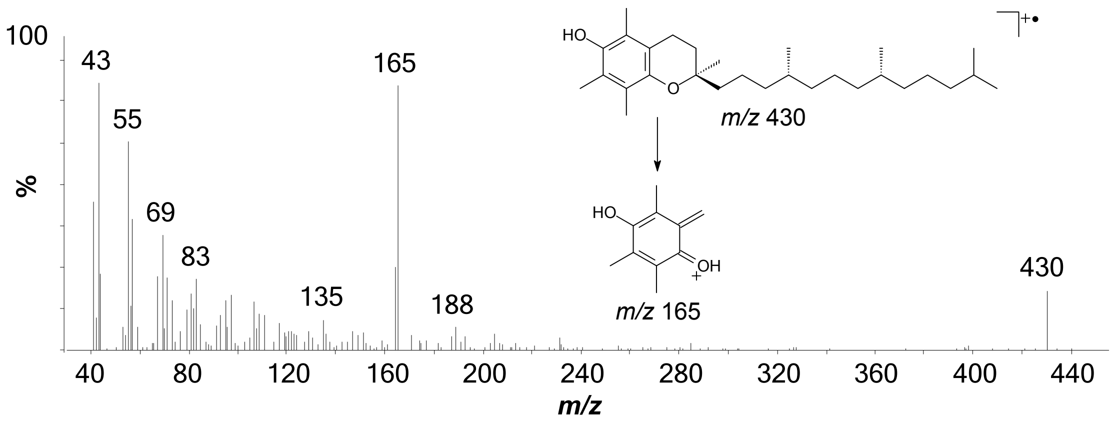

Figure S3. Mass spectra of α-tocopherol (EIMS [$M^{+\bullet}$]: 430) and assignment of the ion fragment *m/z* 165 [$C_{10}H_{13}O_2$]$^+$.

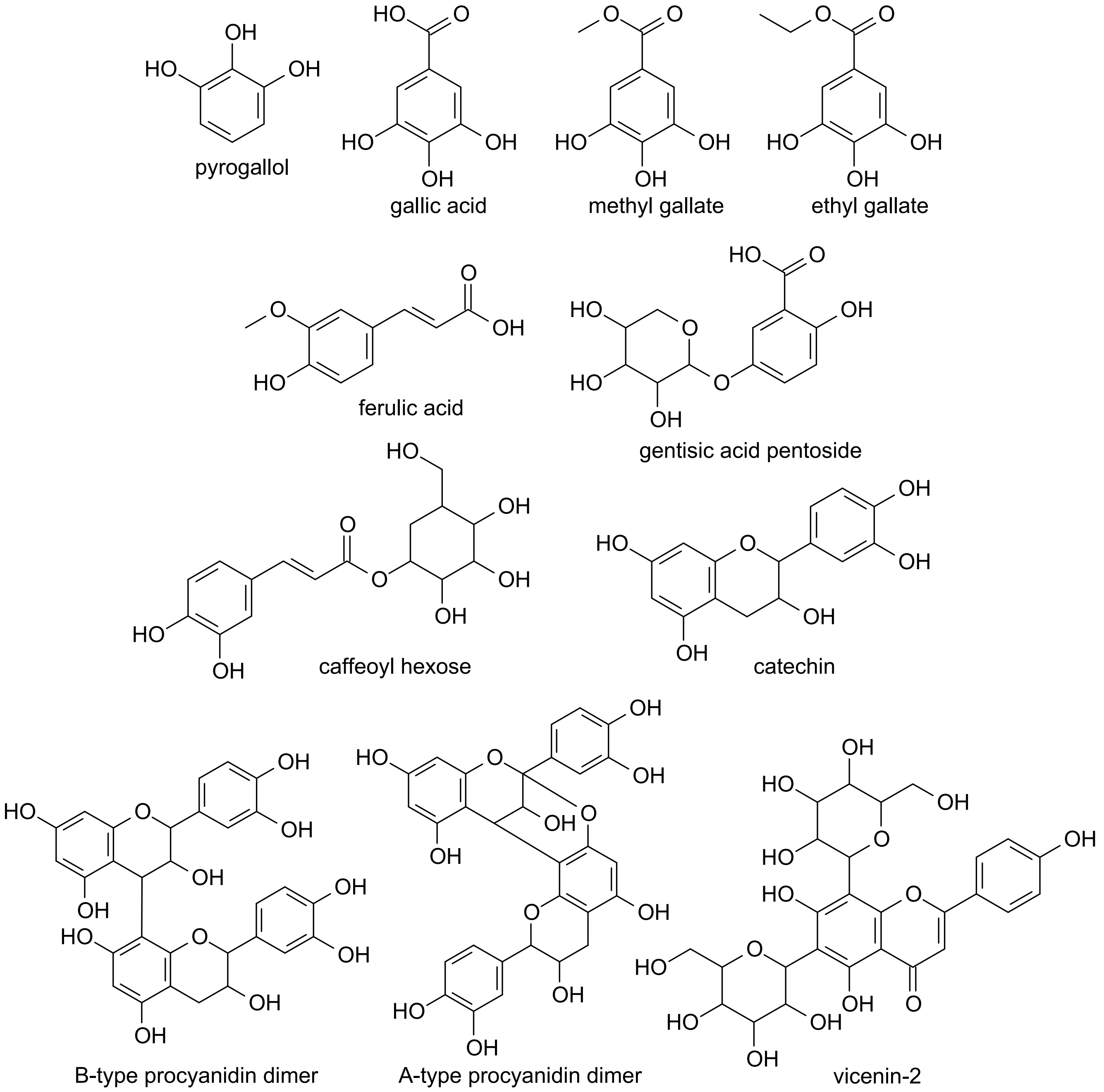

Figure S4. Chemical constituents identified in the EtOH extract of *Mimosa caesalpiniifolia* stem bark by ESI(−)-LTQ-Orbitrap-MS.
